# The effect of moderate physical activity on NK cells populations and cytotoxic T lymphocytes in young, healthy women

**DOI:** 10.1371/journal.pone.0349215

**Published:** 2026-05-29

**Authors:** Anna Tubek-Krokosz, Jarek Baran, Andrzej Galbarczyk, Adrianna Gałuszka-Bulaga, Magdalena Klimek, Karolina Krzych-Miłkowska, Karolina Leś, Kinga Słojewska, Monika Ścibor, Grazyna Jasienska

**Affiliations:** 1 Department of Environmental Health, Faculty of Health Sciences, Jagiellonian University Medical College, Krakow, Poland; 2 Jagiellonian University Medical College, Doctoral School of Medical and Health Sciences, Krakow, Poland; 3 Department of Clinical Immunology, Institute of Pediatrics, Jagiellonian University Medical College, Krakow, Poland; 4 Department of Clinical Immunology, University Children’s Hospital, Krakow, Poland; 5 Lise Meitner Research Group BirthRites - Cultures of Reproduction, Max Planck Institute for Evolutionary Anthropology, Leipzig, Germany; University of Miami, UNITED STATES OF AMERICA

## Abstract

**Background:**

Physical activity is a modifiable lifestyle factor known to reduce the risk of many diseases, including cancer, partly through improvement of immune function. Physical exercise activates innate and adaptive immunity, boosting the circulation and function of NK cells and cytotoxic T lymphocytes, key players in cancer surveillance. This study aims to assess the effect of moderate physical activity on NK cells, their subpopulations, and cytotoxic T lymphocytes, in young, healthy women.

**Methods:**

The study included 141 healthy women aged 20–36. Physical activity was monitored using Fitbit accelerometers over two menstrual cycles, during which participants performed at least 180 minutes/week of moderate physical activity. Blood samples were collected to assess cytotoxic T lymphocytes, NK cells, their subpopulations, and NK cytotoxic activity via flow cytometry. Associations between physical activity and immune parameters, adjusting for age and body fat percentage, were analyzed by multiple linear regression models.

**Results:**

A higher number of steps per day was positively associated with the proportion of CD56^bright^CD16^+^ NK cells (β = 0.22, p = 0.016), and minutes spent fairly active per day showed a positive association with CD56^bright^CD16^−^ NK cells (β = 0.17, p = 0.051). Higher number of very active minutes per day was associated with stronger natural killing ability of NK cells (β = 0.27, p = 0.030) and with a smaller percentage change between natural and IL-2-activated cytotoxicity (β = −0.25, p = 0.048). No significant associations were observed for total NK cells or cytotoxic T lymphocytes.

**Conclusions:**

A longer-term moderate physical activity in healthy young women may enhance CD56^bright^ NK cell subpopulations and natural NK cell cytotoxicity, while reducing responsiveness to IL-2 stimulation. Our findings provide insight into a better understanding of how physical activity affects immune functioning, which might be relevant for cancer prevention, including breast cancer, in healthy women.

## Introduction

Physical activity is a modifiable lifestyle factor reducing the risk of numerous diseases, including cancer, and in particular breast cancer in women [1–5]. Physical activity has also been found to reduce the side effects of cancer treatment, prolong survival, and improve patients’ quality of life [1,6,7]. It is hypothesized that one of the most important mechanisms of the protective anti-cancer effect of physical activity is the improvement of immune function, including the reduction of inflammation [8–12].

The immune system is highly responsive to exercise. Physical activity generates a systemic response that affects both the innate and adaptive immune systems [13]. This response enhances immune surveillance by increasing the circulation and functional capacity of immune cells, such as natural killer cells (NK cells) and cytotoxic T lymphocytes, thereby reducing the risk of cancer development [14,15].

NK cells, which participate in the first line of innate immune defense, play a crucial role in the early response against tumors and viral infections by recognizing and eliminating transformed or virus-infected cells without prior sensitization. They exert cytotoxicity through the release of perforin and granzymes, which induce apoptosis in virus-infected or malignant cells [16–18]. NK cells are broadly divided into two major subpopulations based on the surface expression of CD56: CD56^bright^ and CD56^dim^ subsets. The CD56^bright^ subset, found mainly in secondary lymphoid tissues, is immunoregulatory and produces high levels of cytokines. In contrast, CD56^dim^ NK cells dominate in peripheral blood (~90%) and possess strong cytotoxic activity, eliminating target cells via cytolytic granules and antibody-dependent cellular cytotoxicity (ADCC) [16,19].

Complementing this innate response, cytotoxic T lymphocytes are critical components of the adaptive immune system. They also eliminate virus-infected and cancer cells by releasing perforin and granzymes, leading to apoptosis in their targets [20]. Through these mechanisms, cytotoxic T lymphocytes are essential not only for controlling infections but also for suppressing tumor development and maintaining immune surveillance [21].

Together, NK cells and cytotoxic T lymphocytes provide a coordinated defense strategy, with NK cells offering rapid, nonspecific cytotoxicity and cytotoxic T lymphocytes delivering antigen-specific responses critical for long-term immune protection.

Intensive and moderate physical activity exerts differential effects on the immune system, particularly on NK cells and cytotoxic T lymphocytes. Intensive (prolonged or high exertion) exercise can induce temporary immunosuppression. Although it initially increases the number of NK cells and cytotoxic T lymphocytes in the bloodstream due to stress-related mobilization, their numbers and activity may decline post-exercise. This might be linked to increased levels of cortisol and other stress hormones, which suppress immune function [13,22].

Moderate exercise, on the other hand, is associated with enhanced immune surveillance. It leads to a transient increase in circulating NK cells and cytotoxic T lymphocytes, followed by improved trafficking and function. NK cells show increased cytotoxicity, while cytotoxic T lymphocytes demonstrate improved responsiveness and proliferation [13,23].

Notably, NK cells demonstrate rapid and pronounced sensitivity to acute changes in exercise intensity, with fluctuations in their circulation occurring more immediately [13]. In comparison, cytotoxic T lymphocytes are more affected in terms of their functional capacity and proliferation over a longer timescale, rather than through immediate changes in cell count [15].

Acute physical exercise is well-studied in terms of the immune system response, particularly the mobilization of NK cells, in both healthy athletic and nonathletic individuals [24–32]. Among studies examining the impact of moderate physical activity on NK cells and/or cytotoxic T lymphocytes in women, the majority focused on breast cancer patients [33–35], breast cancer survivors [36,37], women with other health conditions [38,39], and middle-aged [40,41] or older women [42,43]. Only a few studies included young and generally healthy women [44,45].

Even though breast cancer is the main cancer affecting women [46] and physical activity has well documented risk reducing impact [1–5], the systemic immune response to moderate physical activity in young, healthy women is still not well understood. Deepening the knowledge in this area is essential for developing effective breast cancer prevention strategies. Therefore, the purpose of this study was to assess the effect of long-term moderate physical activity on NK cells and their subpopulations, as well as cytotoxic T lymphocytes, in young, healthy women. NK cell cytotoxicity was also examined. Importantly, the study took into account participants’ total physical activity, encompassing recreational activities, sports and exercise, household chores, occupational activities, walking, and other forms of non-exercise-related movement measured over two months, allowing for a reliable assessment of long-term total activity levels.

## Materials and methods

The present study was part of a larger research project investigating the role of physical activity in breast cancer prevention. The research project complied with the ethical standards specified in the Declaration of Helsinki and was approved by the Bioethics Committee of the Jagiellonian University (decision number: 1072.6120.47.2018 from April 20, 2018).

### Participants

The participants of the study were women living in Krakow, Poland. Women were recruited through local and social media advertisements across three recruitment rounds: the first from February 11, 2019, to April 16, 2019; the second from November 12, 2019, to March 10, 2020; and the third from October 18, 2022, to October 9, 2023. The first stage of qualification for the study was a telephone interview in order to verify the inclusion criteria, which involved age between 20 and 35 years, non-smoking status, and the absence of any health contraindications to undertaking physical activity. The final decision on inclusion in the study was made after a consultation with a medical doctor who was a member of the research team. Out of 234 women who took part in the research project, 93 participants were not included in the present analysis: 64 women did not provide blood samples; 5 participants did not provide accelerometer (used for monitoring physical activity) data, either due to failure to wear the device or issues with data recording; 2 women had incomplete accelerometer data; 2 women had abnormalities identified through a complete blood count (one case of lymphocytosis and one of eosinophilia); 9 women had abnormal NK cell levels, either below or above the reference range, 1 woman had an abnormal result for the NK subpopulation level; 1 woman had an abnormal level of cytotoxic T lymphocytes, exceeding the reference range; and 9 women were taking antidepressant medications. Therefore, the final study sample included 141 women. All recruited women provided written informed consent to participate in the study after being informed about the objectives and requirements of the study.

### General questionnaire and anthropometric measurements

Women who met the study inclusion criteria were invited to a meeting. Each participant was interviewed by a trained research assistant. A general questionnaire was used to collect demographic, lifestyle, and overall health data. Participants’ body height was measured by a manual anthropometer, with no shoes. Body weight and body fat percentage were assessed by bioimpedance analysis using Tanita BC-545N scale (Tokyo, Japan), with participants standing barefoot on the scale.

### Physical activity

Women took part in the project for three consecutive menstrual cycles. During cycles 2 and 3, women were requested to increase their physical activity to at least 180 minutes of moderate to vigorous physical activity per week. The minimum requirements for increased physical activity in our study have been established based on World Health Organization (WHO) guidelines [47]. In the present study, we analyzed physical activity from these two menstrual cycles. Each participant received a pass to a chain of gyms and fitness clubs, which allowed for unlimited access to training sessions and the choice of activities according to preferences. Physical activity data were collected using Fitbit Alta HR wristband accelerometers (Fitbit, Inc.; San Francisco, CA, USA). Participants were asked to wear Fitbit device on their preferred wrist 24 hours a day (except while bathing, showering, or swimming). The settings in accelerometers have been personalized for each woman (dominant hand, sex, age, body height, and weight). Fitbit devices were also programmed to send notifications after extended periods of inactivity to support participants in adhering to recommendations for increasing physical activity. The accelerometer provided data on daily steps taken, minutes spent being active, and total energy expenditure. Active minutes estimates calculated by Fitbit device are based on metabolic equivalents (METs) and are divided into three categories: minutes spent lightly active (activity intensity <3 METs), minutes spent fairly active (3–6 METs), and minutes spent very active (>6 METs). Total energy expenditure calculated by the accelerometer is the sum of the basal metabolic rate (BMR) and energy used for all activities. The data was downloaded from Fitbit servers using a dedicated application in which an account was set up for each participant.

The Fitbit device readings were considered valid if the accelerometer was worn for at least 7 days during a given menstrual cycle. Previous studies suggest that a minimum of 5 measurement days is needed to reliably estimate monthly daily step count levels in adults using wrist-based trackers [48]. Using the available data, we calculated the average number of steps per day, the average minutes spent lightly, fairly, and very active, and the average total energy expenditure for each woman during each menstrual cycle.

### Blood sampling and hematology analysis

Blood samples were collected after the third menstrual cycle was completed. Each woman informed a member of the research team when she began and ended each cycle. A date for blood collection was then set within two weeks.

Peripheral blood samples collected on EDTA were immediately used for flow cytometric analysis of lymphocyte and NK cell subpopulations, as well as for assessment of NK cell cytotoxic function.

#### Detection of cytotoxic T lymphocytes and NK cells.

EDTA-anticoagulated peripheral blood samples were stained with a panel of fluorescently conjugated monoclonal antibodies using the BD Multitest 6-Color TBNK reagent (anti-CD3-FITC/anti-CD16-PE + anti-CD56-PE/anti-CD45-PerCP-Cy5.5/anti-CD4-PE-Cy7/anti-CD19-APC/anti-CD8 APC-Cy7; BD Biosciences, Milpitas, CA, USA). Samples were incubated for 15 minutes in the dark at room temperature. Following staining, red blood cells were lysed using BD FACS Lysing Solution (BD Biosciences), and flow cytometry analysis was performed using a 10-color FACS CantoX flow cytometer and BD FACSDiva software version 8.0.1 (BD Biosciences) on a gated population of lymphocytes (S1 Fig). Absolute numbers of cytotoxic T lymphocytes (CD3 ⁺ CD8⁺) and NK cells (CD3 ⁻ CD16 ⁺ /CD56⁺) were determined based on a total lymphocyte cell count.

#### Detection of NK cell subsets.

EDTA-anticoagulated peripheral blood samples were stained with fluorescently conjugated monoclonal antibodies, containing anti-CD45-APC-H7, anti-CD3-FITC, anti-CD56-PE-Cy7, anti-CD16-BV605, and anti-CD19-BV510 (all from BD Biosciences). Following 15 minutes of incubation in the dark at room temperature, erythrocytes were lysed using BD FACS Lysing Solution (BD Biosciences), and samples were subsequently washed with phosphate-buffered saline (PBS). Flow cytometry analysis was performed using a 10-color FACS CantoX flow cytometer and BD FACSDiva software version 8.0.1 (BD Biosciences). NK cell subsets were analyzed after gating blood leukocytes on CD45^+^CD3^-^CD19^-^ cells, according to CD56 and CD16 co-expression (S2 Fig). Absolute numbers of NK cell subsets were calculated relative to the total lymphocyte count.

#### NK cell cytotoxicity assay.

The cytotoxic activity of NK cells was assessed in 74 participants included in the analysis. The results from one participant were excluded due to an atypical response pattern (lack of activation increase following interleukin 2 (IL-2) stimulation). Consequently, NK cell cytotoxicity was evaluated in a total of 73 participants.

NK cytotoxitc activity test was performed using an NKtest (Celonic, Heidelberg, Germany), according to manufacturers’ protocol. Briefly, peripheral blood mononuclear cells (PBMCs) were isolated from heparin-treated peripheral blood of individual study participants by standard Pancoll human (Panbiotech, Aidenbach, Germany) density-gradient centrifugation and resuspended in 0.5 ml of medium provided by the manufacturer. K562 cells were thawed and resuspend in manufacturers’ medium and adjusted to concentration 1x10^5^ cells per milliliter. PBMCs and K562 cells were mixed in a ratio 50:1 in two tubes: 1) unstimulated probe, 2) probe with IL-2 (6 U/sample). Cells were cocultured for 120 min in a humidified CO_2_ incubator. K562 cells alone (or in the presence of IL-2 only) were incubated in parallel and used to assess the spontaneous target cell death (control samples). After the incubation, the cells were placed on ice until flow cytometry analysis. Before acquisition, 50µl DNA staining solution was added to the probes in order to distinguish dead cells. The natural and IL-2-induced NK-cell cytotoxicity was calculated after subtracting the percent of dead K562 cells detected in the control samples from unstimulated and IL-2-stimulated probes, respectively.

The percentage change in cytotoxic activity between unstimulated cells (natural) and IL-2-activated was also calculated (ΔCytotoxicity = (% of IL-2 induced cytotoxicity - % of natural cytotoxicity)/ % of natural cytotoxicity x 100%).

### Statistical analyses

Multiple linear regression models were used to examine the relationships between physical activity and immune parameters, including the percentages of cytotoxic T lymphocytes (CD3^+^CD8^+^), total NK cells (CD3^-^CD56^+^), NK cell subpopulations (CD56^bright^CD16^-^, CD56^bright^CD16^+^, and CD56^dim^CD16^+^), and the cytotoxic activity of NK cells. Physical activity was assessed using several metrics: number of steps per day, minutes spent lightly, fairly, and very active per day, and total energy expenditure (kcal/day), averaged across menstrual cycles 2 and 3, during which participants were instructed to increase their activity levels.

All models were adjusted for potential confounding variables, depending on the analysis. Age and body fat percentage were included in the models involving physical activity measures expressed in steps/day and minutes/day, while age alone was included in models using total energy expenditure. For immune parameters and the cytotoxic activity of NK cells exhibiting a positively skewed distribution, the data were log-transformed using the natural logarithm (ln). Results were considered statistically significant at p ≤ 0.05.

Analyses were conducted separately for immune cell percentages (n = 141) and for NK cell cytotoxic activity measures (n = 73), including unstimulated (natural) and IL-2-induced cytotoxicity, and percentage change in this activity (ΔCytotoxicity). All statistical analyses were performed using IBM SPSS Statistics 29 for Windows.

## Results

Characteristics of the study participants, their physical activity, and immune parameters are presented in Table 1. The number of observations for individual variables may vary slightly due to missing data, except for the assessment of NK cell cytotoxic activity, which was conducted in a smaller group of participants.

**Table 1:** Demographic and anthropometric characteristics, physical activity levels, and immune parameters of the study participants.

	n	Mean (SD)	Min	Max
Age (years)	141	26.7 (4.4)	20	36
Body height (cm)	141	165.5 (6.0)	146.0	183.8
Body weight (kg)	139	60.5 (8.6)	41.1	87.0
Body fat (%)	139	24.9 (6.4)	12.3	41.1
Number of steps per day^a^	141	10472.2 (2679.7)	3763.8	18331.8
Minutes spent lightly active per day^a^	140	245.8 (54.0)	127.3	448.5
Minutes spent fairly active per day^a^	140	26.6 (13.4)	1.9	77.0
Minutes spent very active per day^a^	140	25.7 (15.7)	3.5	87.4
Total energy expenditure (kcal/day)^a^	141	2205.9 (267.9)	1554.1	2966.0
Cytotoxic T lymphocytes, CD3 ^+^ CD8^+^ (% of T cells)	141	23.50 (4.65)	12.10	35.90
NK total cells, CD3^-^CD56^+^ (% of total lymphocytes)	141	14.15 (6.10)	5.50	30.30
NK subset CD56^bright^CD16^-^ (% of total NK cells)	141	4.19 (2.60)	0.50	14.50
NK subset CD56^bright^CD16^+^ (% of total NK cells)	141	3.81 (2.33)	0.40	15.75
NK subset CD56^dim^CD16^+^ (% of total NK cells)	141	81.98 (7.46)	60.68	94.10
Unstimulated (natural) cytotoxic activity of NK cells (% of dead target K562 cells in a co-culture with PBMC - % of dead K562 cells cultured alone)	73	21.46 (13.36)	1.61	63.60
IL-2-activated cytotoxic activity of NK cells (% of dead target K562 cells in a co-culture with PBMC and IL-2 - % of dead K562 cells cultured with IL-2 alone)	73	36.78 (17.55)	4.60	82.40
ΔCytotoxicity (%)^b^	73	92.81 (56.28)	5.96	253.03

Abbreviations: SD, standard deviation; NK, natural killer; PBMC, peripheral blood mononuclear cells; IL-2, interleukin 2

^a^ data averaged across menstrual cycles 2 and 3 (participants increased activity levels during these cycles)

^b^ percentage change in NK cell cytotoxic activity between unstimulated (natural) and IL-2-activated conditions

Analysis of NK cell subpopulations revealed a significant positive association between the number of steps per day and the proportion of CD56^bright^CD16^⁺^ NK cells (β = 0.22, p = 0.016) (Table 2). Additionally, a significant positive association was observed between minutes spent fairly active per day and the proportion of CD56^bright^CD16^−^ NK cells (β = 0.17, p = 0.051). There were no statistically significant associations between levels of physical activity and the percentage of cytotoxic T lymphocytes or total NK cells (p values varied from 0.293 to 0.735), nor were any additional significant associations detected for other NK cell subpopulations.

**Table 2:** Relationships between cytotoxic T lymphocytes, NK cells, and their subpopulations, and cytotoxic activity of NK cells with physical activity in women. Results from multiple regression models.

	Number of steps per day*			Minutes spent lightly active per day*			Minutes spent fairly active per day*			Minutes spent very active per day*			Total energy expenditure (kcal/day)**		
	β	SE	p	β	SE	p	β	SE	p	β	SE	p	β	SE	p
**Cell population (%), n = 141**															
Cytotoxic T lymphocytes (CD3 ^+^ CD8^+^ out of CD3^+^)	−0.05	0.00	0.564	0.05	0.01	0.604	−0.04	0.03	0.625	−0.06	0.03	0.537	−0.03	0.00	0.735
NK total cells (CD3^-^CD56^+^ out of total lymphocytes)	−0.04	0.00	0.630	0.07	0.00	0.424	−0.09	0.00	0.293	0.05	0.00	0.555	−0.09	0.00	0.320
NK subset CD56^bright^CD16^-^ (out of total NK cells)	0.11	0.00	0.234	0.02	0.00	0.806	0.17	0.00	**0.051**	0.03	0.00	0.781	0.04	0.00	0.667
NK subset CD56^bright^CD16^+^ (out of total NK cells)	0.22	0.00	**0.016**	0.04	0.00	0.636	0.12	0.00	0.170	0.14	0.00	0.121	0.05	0.00	0.568
NK subset CD56^dim^CD16^+^ (out of total NK cells)	−0.01	0.00	0.941	0.07	0.01	0.432	−0.09	0.05	0.289	−0.01	0.04	0.870	0.05	0.00	0.558
**Cytotoxic activity of NK cells (%), n = 73**															
Unstimulated (natural) (% of dead target K562 cells in a co-culture with PBMC - % of dead K562 cells cultured alone)	0.09	0.00	0.472	−0.16	0.00	0.181	0.09	0.01	0.456	0.27	0.01	**0.030**	−0.16	0.00	0.190
IL-2-activated (% of dead target K562 cells co-cultured with PBMC and IL-2 - % of dead K562 cells cultured with IL-2 alone)	0.14	0.00	0.273	−0.03	0.04	0.833	0.09	0.16	0.476	0.21	0.14	0.089	−0.15	0.01	0.208
ΔCytotoxicity^a^	−0.06	0.00	0.658	0.19	0.00	0.110	0.01	0.01	0.945	−0.25	0.00	**0.048**	−0.02	0.00	0.847

Note: Boldface indicates statistical significance (p ≤ 0.05)

Abbreviations: NK, natural killer; PBMC, peripheral blood mononuclear cells; IL-2, interleukin 2

^a^ percentage change in NK cell cytotoxic activity between unstimulated (natural) and IL-2-activated conditions

*Adjusted for age and body fat

**Adjusted for age

Regarding NK cell cytotoxicity, minutes spent very actively per day were significantly, positively associated with unstimulated (natural) cytotoxic activity of NK cells (β = 0.27, p = 0.030) and significantly negatively associated with ΔCytotoxicity – percentage change in NK cell cytotoxicity between unstimulated (natural) and IL-2-activated conditions (β = −0.25, p = 0.048). No other significant relationships were found for NK cell cytotoxic activity across other physical activity metrics evaluated. The results of the analyses of cytotoxic T lymphocytes, NK cells, and their subpopulations, and the cytotoxic activity of NK cells with physical activity in women are presented in Table 2.

## Discussion

In this study, we investigated the effects of long-term moderate physical activity on key immune cell populations involved in anti-tumor responses, such as NK cells and cytotoxic T lymphocytes in healthy young women. Additionally, we assessed NK cell cytotoxicity under both natural conditions and following stimulation with IL-2. Physical activity was assessed using several metrics: number of steps per day, minutes spent lightly, fairly, and very active per day, and total energy expenditure (kcal/day). In this context, a strong association was observed within a specific subpopulation of NK cells. Higher daily steps were significantly associated with a greater proportion of the CD56^bright^CD16^+^ NK cell subpopulation. Moreover, a significant positive association was found between minutes spent fairly active per day and the proportion of CD56^bright^CD16^−^ NK subpopulation.

CD56^bright^ NK cells encompass functionally distinct subsets defined by their level of CD16 co-expression. CD56^bright^CD16^−^ NK cells are characterized by high CD56 expression and little to no CD16 co-expression. Predominantly located in lymphoid tissues, these cells are known for their potent cytokine production rather than direct cytotoxic activity [49,50]. Notably, they may act as precursors to the more cytotoxic CD56^dim^ NK cell subset [49,51]. An increased proportion of CD56^bright^CD16^−^ NK cells has been associated with enhanced immunoregulatory capacity and more effective control of inflammatory responses [50,51]. Their expansion in response to minutes spent fairly actively per day may therefore reflect a potentially beneficial immunomodulatory adaptation, supporting tissue integrity and balanced immune function. In contrast, CD56^bright^CD16^+^ NK cells represent a transitional subset with intermediate functional characteristics. While retaining high CD56 expression, these cells express low to moderate levels of CD16, which endows them with a greater potential for ADCC compared to their CD16^−^ counterparts. Functionally, CD56^bright^CD16^+^ NK cells combine robust cytokine secretion with an increased cytotoxic capacity, bridging the gap between purely regulatory CD56^bright^CD16^−^ NK cells and the highly cytotoxic CD56^dim^ NK population. These cells are found both in peripheral blood and secondary lymphoid tissues, suggesting a role in both systemic immune surveillance and tissue-specific immune responses. An increased proportion of CD56^bright^CD16^+^ NK cells, in response to taking more steps per day, could enhance both immunoregulatory and cytotoxic responses, contributing to more effective early defense against pathogens while maintaining balanced immune function [50,51]. The earlier research [44] analyzing NK cell subpopulations in healthy women demonstrated a significant decrease in the level of CD56^bright^CD16^dim^ subset (in our study, corresponds to the CD56^bright^CD16^+^ subset) following a single bout of moderate-intensity exercise, with an even greater reduction of its proportion observed after high-intensity exercise. This previous study focused on the acute effects of physical exertion and showed that exercise intensity distinctly affects NK cell subset mobilization, indicating that acute and chronic physical activity may exert divergent effects on NK cell dynamics and immune regulation.

In our study, we did not observe any significant associations between physical activity and the percentage of total NK cells or cytotoxic T lymphocytes. In contrast, the previous study [44] demonstrated that physical exercise, both moderate and intense, led to increases in the percentage of NK cells, highlighting that the type, intensity, and duration of exercise play a crucial role in modulating the composition of immune cell populations. Additionally, another study [45] investigating an 8-week aerobic dance exercise intervention reported significant increases in the absolute count of NK cells as well as cytotoxic T lymphocytes in the exercise group. This difference may be due to the fact that the described study measured absolute cell counts, whereas our study focused on percentage values.

Importantly, in our study, we observed significant associations between physical activity and NK cell cytotoxic function. Minutes spent very active per day were positively associated with the unstimulated (natural) cytotoxicity of NK cells, suggesting that higher-intensity exercise may enhance innate immune readiness [13,14]. It is known that acute and chronic physical exercise can boost NK cell activity, potentially contributing to improved immune surveillance [15]. Moreover, a recently published review [52] confirmed that both acute and chronic physical activity can promote NK cell activation and cytotoxic potential, thereby supporting exercise as a non-pharmacological immunomodulatory strategy. High-intensity exercise has also been previously shown to induce a more pronounced increase in NK cell activity than moderate-intensity efforts, indicating a dose-response relationship between exercise intensity and the activation of the innate immune system [53]. Additionally, we observed that minutes spent very actively per day were also negatively associated with the percentage change between unstimulated (natural) and IL-2-activated NK cell activity. This may indicate that women engaging in higher-intensity activity exhibit less relative enhancement upon cytokine stimulation, potentially because their baseline cytotoxic function is already elevated. Such a pattern is consistent with the notion that regular vigorous physical activity primes NK cells for immediate cytotoxic action, reducing the magnitude of further activation required [54,55]. Specifically, a recent study [55] reported that high-intensity exercise enhances NK cell mobilization and cytotoxic function.

Our findings in healthy young women are consistent with previous studies in breast cancer patients undergoing chemotherapy [33–35], where NK cell numbers remained stable while their functional activity increased. Similar patterns have also been reported in older or healthy women following moderate or structured exercise interventions [40–45]. Together, these observations suggest that functional activation of NK cells can occur independently of total cell counts, emphasizing the importance of assessing immune cell function rather than quantity of cells alone. Differences between studies likely reflect variations in exercise type, intensity, and duration. Nonetheless, our results extend these observations by showing that even in young, healthy women, long-term moderate-to-vigorous daily physical activity can enhance NK cytotoxic function and modulate subpopulation distribution, potentially priming the innate immune system for more efficient responses to pathogens or transformed cells. These observations highlight that functional enhancements in NK cells, even without changes in their total number, may represent a biologically meaningful adaptation of the immune system. Given the central role of NK cells in early antiviral responses and tumor immunosurveillance [17,19], such improvements could contribute to mechanisms that support cancer prevention, including breast cancer. However, it should be emphasized that the present study was not designed to assess clinical outcomes; therefore, it cannot be determined to what extent these immunological changes may translate into measurable health benefits, such as reduced infection risk or long-term disease prevention. Nevertheless, our findings support the concept that regular physical activity contributes to the optimization of immune function even in a young and generally healthy women, in whom baseline immune competence is expected to be high.

The study has some strengths and limitations. An important strength of our study was the assessment of the impact of physical activity on NK cell subpopulations, rather than concentrating solely on the total NK cell population, as was commonly done in previous research. This approach is particularly important, as NK cell subpopulations differ in their functional properties and may respond differently to physical activity, which could have important implications for understanding immune regulation and developing targeted interventions. Another aspect contributing to the innovative nature of the study was the inclusion of physical activity assessment, not only the intentional exercise but all daily activities, such as physical work and walking. Participants’ physical activity levels were monitored on a daily basis over two menstrual cycles, constituting a long-term assessment. We focused on cycles 2 and 3, during which participants were instructed to increase their physical activity to at least 180 minutes per week of moderate to vigorous activity in line with WHO guidelines [47]; these cycles therefore reflect the period of adherence to the prescribed activity intervention and allow evaluation of its effects on immune parameters. Additionally, physical activity was assessed using various indicators. This comprehensive approach provides a more accurate representation of real-life activity patterns and their potential impact on immune function. Finally, a significant strength of the study was the inclusion of a relatively homogeneous group of young, healthy women, contrary to the previous studies that primarily focused on women with various medical conditions, including cancer, or on older populations. Although the homogeneity of the study group may limit the generalizability of the findings, it offers valuable insights into the immune response within a well-controlled, generally healthy population.

Although Fitbit wristband accelerometers provide a convenient and objective method for monitoring physical activity and are widely adopted [56,57], their measurements may involve some degree of variability. This constitutes a limitation of our study. Fitbit device precision depends on correct usage, which can differ between individuals and affect data consistency [58]. Fitbit devices generally provide valid estimates of steps and energy expenditure during basic activities like walking and running [56,59], but they are less effective at capturing non-ambulatory or water-based activities such as cycling or swimming, potentially leading to underestimation of energy expenditure [58].

## Conclusions

Our study provides insights into the relationship between longer-term moderate physical activity and immune cell dynamics in healthy young women. While no significant associations were observed between physical activity and the percentages of total NK cells or cytotoxic T lymphocytes, our findings suggest that physical activity may selectively promote the expansion of specialized NK cell subpopulations, which could have important implications for their anti-tumor activity and overall immune surveillance. Moreover, time spent in vigorous activity during the day was associated with higher natural NK cell cytotoxicity but reduced responsiveness to IL-2 stimulation. These results indicate that physical activity patterns may influence the balance between natural and stimulated NK cell responses. In summary, our findings highlight the potential of long-term moderate physical activity to modulate innate immune function, emphasizing its significance for understanding the immunological mechanisms involved in reducing the risk of cancer. While these findings are relevant to the prevention of many types of cancers and viral infections, we would like to emphasize their potential importance in reducing especially the risk of breast cancer. Although it is well established that physical activity is one of the main modifiable factors in breast cancer prevention [1–5], the mechanisms through which it reduces the risk are not yet fully understood. One of the most commonly postulated mechanisms is the effect of physical activity on reducing lifetime exposures to sex hormones [60], and another one is the improvement of immune function [8–12]. Our results suggest that physical activity, even at relatively moderate levels, may contribute to a reduction in breast cancer risk through immunological mechanisms.

## Supporting information

S1 Fig Gating strategy for flow cytometry analysis of peripheral blood lymphocyte subsets. (PDF)

S2 Fig Gating strategy for flow cytometry analysis of NK cell subsets. (PDF)

S1 Data Dataset. (XLSX)
