# Supplementary figures and images for: The effect of moderate physical activity on NK cells populations and cytotoxic T lymphocytes in young, healthy women
Source: PLoS One. 2026 May 29;21(5):e0349215. doi: 10.1371/journal.pone.0349215 (PMC13221039; doi:10.1371/journal.pone.0349215)

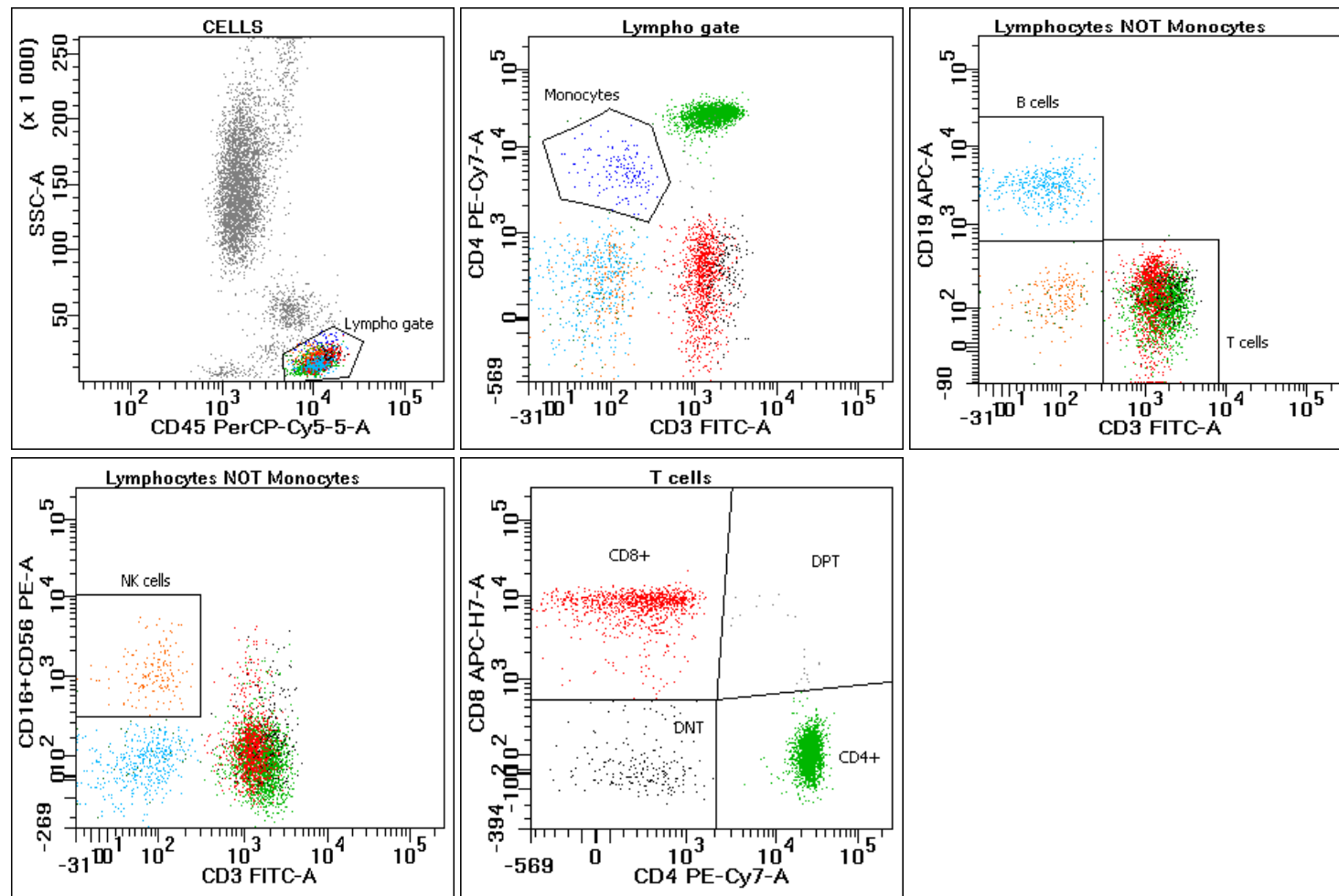

**S1 Fig. Gating strategy for flow cytometry analysis of peripheral blood lymphocyte subsets.**

Supplement: S1 Fig — (PDF) [file pone.0349215.s001.pdf]

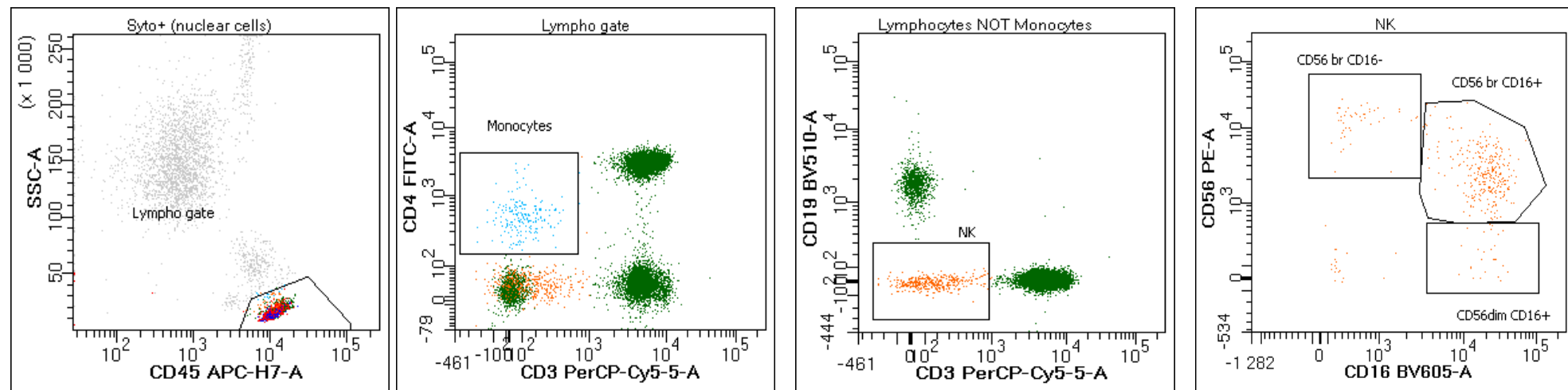

**S2 Fig. Gating strategy for flow cytometry analysis of NK cell subsets.**

Supplement: S2 Fig — (PDF) [file pone.0349215.s002.pdf]
